# Simple Sequence Repeat Fingerprint Identification of Essential-Oil-Bearing *Rosa rugosa* via High-Resolution Melting (HRM) Analysis

**DOI:** 10.3390/biom13101468

**Published:** 2023-09-29

**Authors:** Xiaoyu Xu, Nan Wang, Liguo Feng, Jianwen Wang

**Affiliations:** College of Horticulture and Landscape Architecture, Yangzhou University, Yangzhou 225009, China; 211802232@stu.yzu.edu.cn (X.X.); 2022104094@stu.njau.edu.cn (N.W.)

**Keywords:** SSR, high-resolution melting, *Rosa rugosa*, fingerprint, essential oil

## Abstract

Oil-bearing *Rosa rugosa* are popular in the essential oil and perfume markets. The similar botanical characteristics between high-oil-yield or low-oil-yield cultivars are confusing and it is hard for farmers or breeders to identify the high-oil-yield cultivar by phenotype difference. High-resolution melting (HRM) analysis of simple sequence repeats (SSRs) can construct accurate DNA fingerprints quickly, which was shown to be effective for identification of closely related cultivars of *R. rugosa*. Optimization of HRM-SSR indicated that the 10 µL HRM reaction mixture containing 20 ng of genomic DNA of *R. rugosa* and 0.75 µL of 10 µmol/L of each primer with an annealing temperature of 64 °C was a robust SSR genotyping protocol. Using this protocol, 9 polymorphic SSR markers with 3–9 genotypes among the 19 *R. rugosa* cultivars were identified. The top three polymorphic makers SSR9, SSR12 and SSR19 constructed a fingerprint of all cultivars, and the rare insertion in the flanking sequences of the repeat motif of SSR19 generated three characteristic genotypes of three high-oil-yield cultivars. These results may be economical and practical for the identification of high-oil-yield *R. rugosa* and be helpful for the selection and breeding of oil-bearing roses.

## 1. Introduction

The perfume rose is famous for its widespread use in essential oil extraction [1]. The Middle Eastern *Rose damascena* and the European *Rosa centifolia* are the most well known oil-bearing roses [1,2]. Now their cultivars like ‘Taif Rose’ (*R. damascena* Mill. f. trigintipetala Dieck) and ‘Grasse Rose’ (*Rosa centifolia* cultivar) are planted by Bulgaria, Iran, Morocco and Turkey, contributing most high-quality essential oils to the Europe luxury perfume market [1,2,3]. Though some of these well-known oil-bearing roses were introduced to China several decades ago, cultivars of the East Asian *Rosa rugosa* contribute mostly to the Asian market [4,5]. Pingyin Rose Research Institute of Pingyin, Shandong Provence, China has selected dozens of superior *R. rugosa* individuals or half-sib families via crossbreeding or natural hybridization or introgression since the 1970s, and about 20 oil-bearing cultivars have been identified by specificity, consistency and stability testing and observation [6,7]. Some of the cultivars, e.g., *R. rugosa* ‘Fenghua’ or *R. rugosa* ‘Zizhi’, have been wildly planted in different provinces of China for scented teas and essential oils [8,9]. The oil yields of these cultivars varied between 0.1 and 0.3% and identification of the high-oil-yield cultivars was important [9,10]. Although our previous study has identified eight high-oil-yield cultivars by a DNA marker of the cis-element [10], similar botanical characteristics among high- and low-oil-yield cultivars confuse farmers or breeders [2,11]. It is necessary to construct a fingerprint of oil-bearing cultivars of *R. rugosa* based on DNA markers, not morphological markers.

Some conserved DNA makers like nuclear gene fragment makers (ITS, ITS2) and chloroplast gene fragment markers (matK, rbcL, psbA-trnH) have been used in plant DNA barcodes, although these established markers are not suitable for intraspecific variation [12,13]. RFLP (restriction fragment length polymorphism), RAPD (random amplified polymorphic DNA), AFLP (amplified fragment length polymorphism), single-nucleotide polymorphisms (SNPs) and microsatellites or simple sequence repeats (SSRs) are frequently used markers for fingerprints of cultivars [13,14]. SSRs with high polymorphism, codominance and reproducibility [15] are the most preferred markers for fingerprint construction [16,17]. Gel-based SSR genotyping of PCR amplification using loci-specific primers has been widely used, like polyacrylamide gels or the automated capillary electrophoresis system (CE) [18,19,20]. These gel-based protocols are laborious or costly and powerless for single SNP detection in the flanking sequences of repeat motifs [21]. High-resolution melting (HRM) is a technique based on identifying different melting profiles of post-PCR products [21]. As determined by the real-time measurement of the fluorescence level of double-stranded DNA, HRM is sensitive and effective for variant screening, corresponding to the unique melting curves of DNA fragments [22]. Though it was used in single SNP detection first, the HRM curves were more robust in SSR genotyping due to over 2 bp insertion or detection [23]. Since saturable fluorescence dyes like Eva Green or LC Green have become affordable in recent years, the use of HMR-based SSR genotyping (HRM-SSR) is increasing in plant identification studies [16,21,23].

This study aimed to optimize a robust HRM-SSR protocol for *R. rugosa*. Using the HRM-SSR protocol, we constructed a fingerprint of oil-bearing cultivars using polymorphic markers. The SSR markers and corresponding fingerprint can be a guide for identification of high-oil-yield *R. rugosa* to avoid the misjudgment of high-oil-yield cultivars.

## 2. Materials and Methods

### 2.1. Plant Materials and DNA Isolation

Since the 1980s, the oil-bearing *R. rugosa* cultivars have been collected in the germplasm resource nursery of Pingyin Rose Research Institute along with their preservation, propagation, renewal and identification information. A total of 19 oil-bearing *R. rugosa* cultivars including 8 high-oil-yield cultivars and 11 low-oil-yield cultivars (Table 1) were used in this study. Each 5 g leaf of 3 plants (biological replications) of the 19 cultivars and the wild *R. rugosa* (wild plant, a parent of cultivars) were collected for 3 independent DNA extraction procedures. Total DNA was extracted by a Super Plant Genomic DNA Kit (DP360, Tiangen Biotech Co., Beijing, China) according to the manufacturer’s instructions. DNA concentration was quantified using a spectrophotometer (NanoDrop 2000, Thermo Co., Fremont, CA, USA) and was then adjusted to a 100 ng/µL concentration.

### 2.2. SSR Identification and Evaluation

The genome data of *R. rugosa* were retrieved from GDR (Genome Database for Rosaceae, https://www.rosaceae.org/ (accessed on 1 January 2022)). SSR identification and primer design were based on Krait-v1.3.3 software [24] with default parameters. More than 40,000 loci with di-, tri-, tera-, penta- and hexa-nucleotide motifs of at least 9, 6, 5, 5 and 5 repeats were identified. Sixty putative microsatellite loci distributed in different chromosomes were selected randomly. Only 32 loci (Table S1) producing effective amplification according to the agarose gel electrophoresis with the DNA template of wild *R. rugosa* were selected as candidate SSR markers.

### 2.3. HRM-PCR Amplification and Data Analysis

The 10 µL HRM reaction mixture, including 5 µL of 2× SsoFast Eva Green supermix (Bio-Rad Co., Hercules, CA, USA), 50 ng of genomic DNA and 0.5 µL of 10 µmol/L of both forward and reverse primers were as recommended by manufacturers [25].

SSR-HRM analysis was performed using the CFX96 (Bio-Rad Co.) in a 96-well carousel with the following program: 95 °C for 2 min, 40 cycles of (98 °C for 3 s, annealing temperature for 3 s) and 72 °C for 30 s. After PCR amplification, HRM fluorescence data were collected from 65 °C to 95 °C at 0.5 °C increments with a 0.5 s hold time for each acquisition step. Using normalization (background fluorescence deduction) and temperature shift using Precision Melt Analysis Software 1.3 (Bio-Rad Co.), the plot of the negative derivative of the fluorescence over temperature generated the melting curve profiles.

## 3. Results

### 3.1. SSR-HRM Optimization and Evaluation

**Figure 1:**
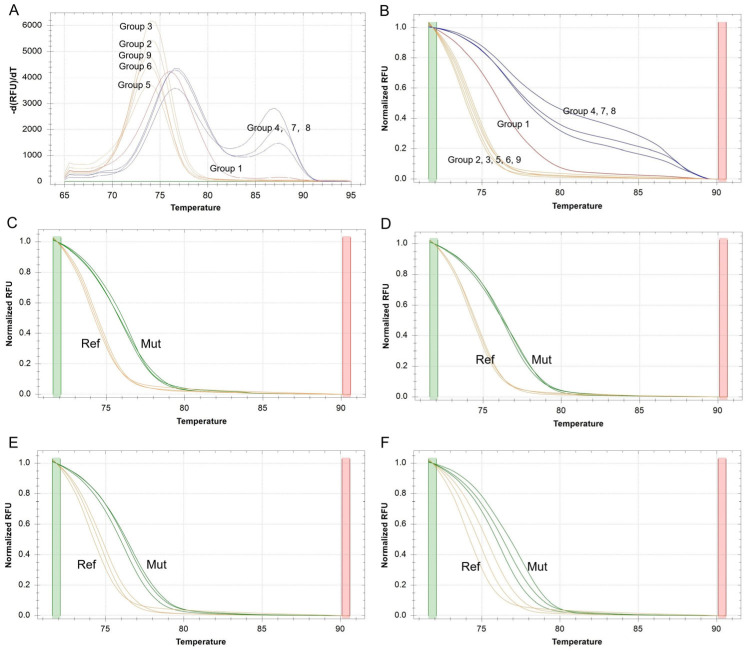
SSR-HRM optimization of orthogonal design groups. (**A**). Derivative plots of melt peak of all groups based on *R. rugosa*. (**B**). Normalized melt curves of all groups based on *R. rugosa*. (**C**–**F**). Normalized melt curves based on *R. rugosa* (Ref, reference genotype) and Pingyin (mut, Mutation genotype) of Groups 2, 3, 5 and 6, respectively.

To develop a robust SSR-HRM protocol, three factors (annealing temperature, DNA concentration and primer concentration) with three levels were optimized using an L9 (3^4^) orthogonal design (Table 2). SSR4 was used for genotyping evaluation due to a previous observation of polymorphism based on the hexamer motif ‘TTTTTG’. In the evaluation of specificity, Groups 2, 3, 5, 6 and 9 generated HRM profiles corresponding to the SSR4 genotype of *R. rugosa* and Group 3 generated the strongest fluorescence signal, while other groups generated two wrong genotypes (Figure 1A,B). The melt peak check indicated that Groups 4, 7 and 8 produced two peaks due to a nonspecific amplification product and Group 1 produced a peak which deviated the right product length (Figure 1A). In the evaluation of reproducibility, three DNA replications of *R. rugosa* and Pingyin each were used for tests of Groups 2, 3, 5, 6 and 9. Groups 2 and 3 generated more accordant curves than Groups 5 and 6 though, they all generated the HRM profiles corresponding to the right genotypes of *R. rugosa* (Figure 1C–F, Ref) and Pingyin (Figure 1C–F, Mut). Group 9 was not discussed in this study due to its incorrect genotyping of Pingyin. The accordant curves among the duplications of Group 3 (more accordant than Group 2) indicated that it could be a robust method for SSR-HRM. By further evaluating the other 18 cultivars, Group 3 maintained a high reproducibility of HRM profiles corresponding to three genotypes (Table S1). In conclusion, the 10 µL HRM reaction mixture amplified at the annealing temperature 64 °C contained 20 ng of genomic DNA and 0.75 µL of 10 µmol/L of each primer would produce robust SSR genotypes of cultivars of *R. rugosa*.

### 3.2. SSR Fingerprint Construction

**Figure 2:**
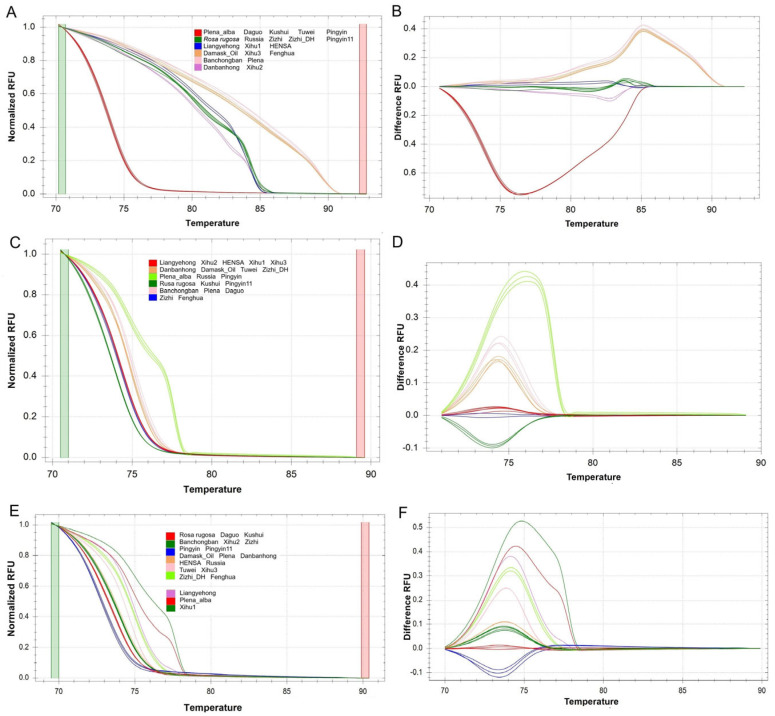
SSR genotyping of 19 *Rosa rugosa* cultivars based on HRM profiles. (**A**,**C**,**E**). Normalized melt curves of SSR9 (SSR 12/19). (**B**,**D**,**F**). Difference curves of SSR9 (SSR 12/19). Genotypes of wild *Rosa rugosa* were used as reference of curves.

From the 32 candidate SSRs, nine loci (SSR4, 9, 11, 12, 13, 19, 28, 29 and SSR31) producing PCR products shorter than 250 bp were selected for SSR-HRM analysis. The nine loci included dimer to hexamer motifs except the trimer. The SSR-HRM analysis of the 19 cultivars and the wild *R. rugosa* produced three (SSR4), four (SSR11, 13, 28), five (29, 31), six (SSR9, 12) and nine (SSR19) genotypes, respectively (Table S1). Genotypes of the most SSRs included at least two cultivars (e.g., SSR9 or SSR12, Figure 2) except for SSR19 (Figure 2E). Three genotypes were markedly different from the other genotypes (Figure 2F).

**Figure 3:**
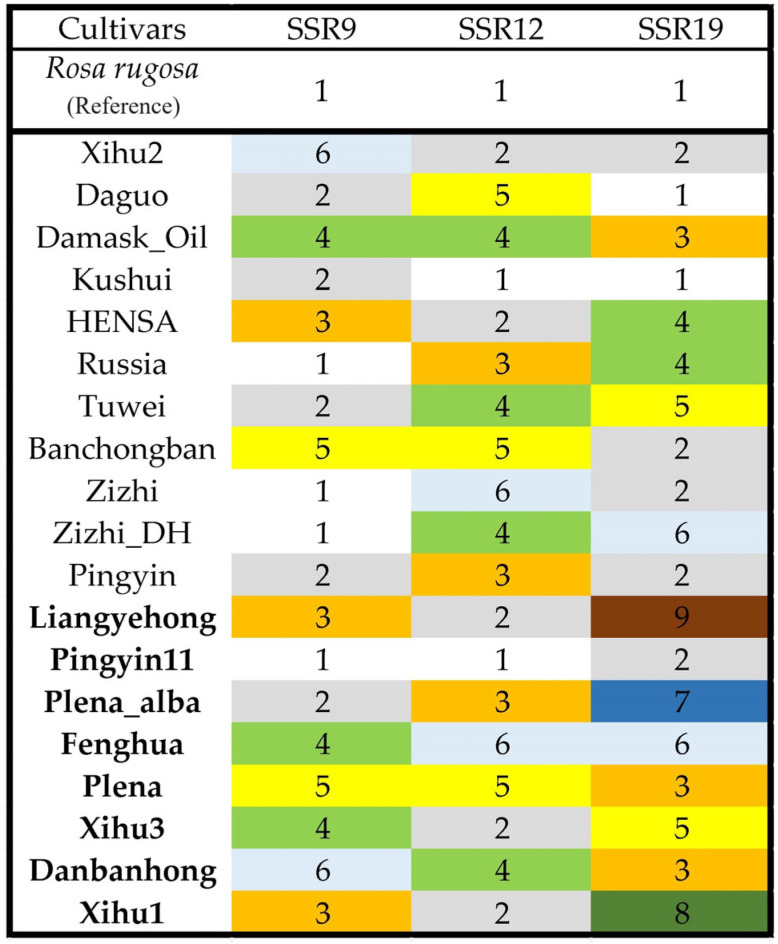
SSR fingerprints of 19 *Rosa rugosa* cultivars. Genotypes of three SSRs were coded following reference genotypes. Bold names indicate the high-oil-yield cultivars.

The genotypes of the top three polymorphic SSRs constructed a DNA fingerprint of all the 19 cultivars (Figure 3). For the eight-oil-yield cultivars, SSR19 could distinguish all them except for Plena and Danbanhong. In particular, codes 7/8/9 could identify Plena_alba, Xihu_1 and Liangyehong with only one SSR marker. We sequenced the three genotypes and checked the variance of sequence length. The motif ‘TCCCG(n)_1–4_TCCCG’ inserted in upstream (Liangyehong) or downstream site (Plena_alba) or both sites (Xihu) of the ‘TCCCG’ repeats and generated longer compound microsatellites.

## 4. Discussion

DNA template, primers and annealing temperature were important for accurate HRM curve profiling [26,27]. Most studies recommended higher annealing temperature for melting curve profiling [26,27,28]. Our optimization of SSR-HRM indicated that a low annealing temperature (56 °C, Groups 1, 4, 7) resulted in nonspecific amplification which generated incorrect SSR genotyping. The DNA amounts of different HRM protocols ranged from 20 to 50 ng in the 10 µL mixture [16,22,26,29]. The excess DNA of *R. rugosa* (100 ng, Groups 7, 8, 9) generated a weaker fluorescence signal and decreased both specificity and reproducibility. For the most commercial HRM mixture containing sensitive Taq (DNA polymerase), the chelation of Mg^2+^ of excess template would inhibit the enzyme activity of Taq.

The extensive hybridization and obscure breeding process of the *Rosa* species resulted in a synonym or homonym among oil-bearing *R. rugosa* or another *Rosa* sp. [4,8,9]. The combination of two SSRs of six genotypes and one SSR of nine genotypes constructed the fingerprint of oil-bearing *R. rugosa*, proving that high polymorphic SSRs were effective for the identification of closely related cultivars in heredity. Besides the genotype combination of polymorphic SSRs, the use of characteristic SSR genotypes is another fingerprint construction method [30]. The three characteristic genotypes of Plena_alba, Xihu_1 and Liangyehong came from the ‘TCCCG(n)_1–4_TCCCG’ insertion in the flanking sequence of SSR19. Unlike the instability of repeat motifs, flanked sequences were regarded as highly conserved [31]. The unconventional insertion sites and variable insertion number of ‘(n)’ indicated that the characteristic genotypes could be rare variants or low-frequent variants [32]. Becoming a longer compound microsatellite explained the high polymorphism of SSR9 to a certain extent [31].

Compared with electrophoresis methods like gel-based genotyping or CE-based genotyping of cultivars of *R. chinensis* [6,11], clover [17], watermelon [33], olive [34] and other relative species [19,20,35], our HRM-SSR of *R. rugosa* cultivars was more efficient, economical and practical. The gel-based SSR fingerprints of *R. chinensis* [6,11], clover [17] and olive [34] were not so persuasive, because the judgement of gel-based genotypes of high polymorphic SSRs was subjective in terms of some content since the SSR bands were mixed with disturbance bands (nonspecific amplification) and showed gel deformation. The indistinguishable SSR genotypes may misadvise fingerprint users. Several CE-based genotyping methods offset the resolution deficiency of gel-based genotyping and identified more polymorphic SSRs for fingerprint construction [19,20], but their cost efficiency was lower than HRM-SSR due to the expensive fluorescent primers and time-consuming nature of the commercial ABI 3730xl platforms [16,23], especially for middle- or low-throughput genotyping. For example, CE-based genotyping (about USD 3) of fingerprint (3 SSRs) of 20 *R. rugosa* is three times the cost of our HRM protocol (about USD 0.5–1 per genotype) without regard to DNA extraction. Additionally, the robustness and automaticity of our HRM-SSR is friendly to oil-bearing rose users, while GE-based genotyping by peak scanning is still dependent on professionals [15,36].

## 5. Conclusions

An Eva Green HRM reaction mixture containing 2 ng/µL of genomic DNA and 37.5 nmol/L of each primer annealed at 64 °C using a CFX96 thermocycler produced robust SSR genotyping of *R. rugosa*. This HRM-SSR protocol identified three polymorphic markers, SSR9, SSR12 and SSR19, which constructed a fingerprint of 19 oil-bearing cultivars. Three characteristic genotypes of SSR19 were specific to three high-oil-yield cultivars. 

## Figures and Tables

**Table 1:** The *Rose rugosa* cultivars used for DNA isolation.

Cultivars	Accession Name ^a^	Botanical Name ^b^
	high-oil-yield cultivars	
1	Plena_alba	*R. rugosa* ‘plena’ f. alba
2	Fenghua	*R. rugosa* ‘Fenghua’
3	Xihu1	*R. rugosa* ‘Xihu1’
4	Xihu3	*R. rugosa* ‘Xihu3’
5	Danbanhong	*R. rugosa* ‘Danbanhong’
6	Liangyehong	*R. rugosa* ‘Liangyehong’
7	Pingyin11	*R. rugosa* ‘Pingyin11′
8	Plena	*R. rugosa* ‘plena’
	low-oil-yield cultivars	
9	XiHu2	*R. rugosa* ‘Xihu2’
10	Pingyin	*R. rugosa* ‘Pingyin’
11	Russia	*R. centifolia* ‘Xiangshui’
12	Banchongban	*R. rugosa* ’Banchongban‘
13	Damask_Oil	*R. damascena* ‘Oil’
14	Kushui	*R. rugosa* ‘Kushui’
15	HANSA	*R. rugosa* ‘HANSA’
16	Daguo	*R. rugosa* ‘Daguo’
17	Zizhi_DH	*R. rugosa* ‘Zizhi_dahong’
18	Zizhi	*R. rugosa* ‘Zizhi’
19	Tuwei	*R. rugosa* ‘Tuwei’

^a^: These accession names are used in the tables and figures of the present work.; ^b^: these cultivars were collected and vouchered by Pingyin Rose Research Institute (Jinan, China).

**Table 2:** The orthogonal design of SSR-HRM optimization of *Rosa rugosa*.

Group	DNA Template (ng)	Tm (°C)	Primers (µL)	Specificity	Reproducibility
1	20	56	0.5	-	-
2	20	60	0.25	B	B
3	20	64	0.75	A	A
4	50	56	0.25	-	-
5	50	60	0.75	B	C
6	50	64	0.5	B	C
7	100	56	0.75	-	-
8	100	60	0.5	-	-
9	100	64	0.25	B	-

‘A, B, C’, the decreasing evaluation of specificity or reproducibility; ‘-’, unspecific amplification or missing data of reproducibility.

## Data Availability

Not applicable.

## Supplementary Materials

The following supporting information can be downloaded at: https://www.mdpi.com/article/10.3390/biom13101468/s1, Table S1: SSR information and primers; Table S2: SSR genotypes by HRM analysis.
